# Supplementary material for: INTS13 variants causing a recessive developmental ciliopathy disrupt assembly of the Integrator complex
Source: Nat Commun. 2022 Oct 13;13:6054. doi: 10.1038/s41467-022-33547-8 (PMC9559116; doi:10.1038/s41467-022-33547-8)
Supplement: Supplementary file 1 — Supplementary Information [file 41467_2022_33547_MOESM1_ESM.pdf]

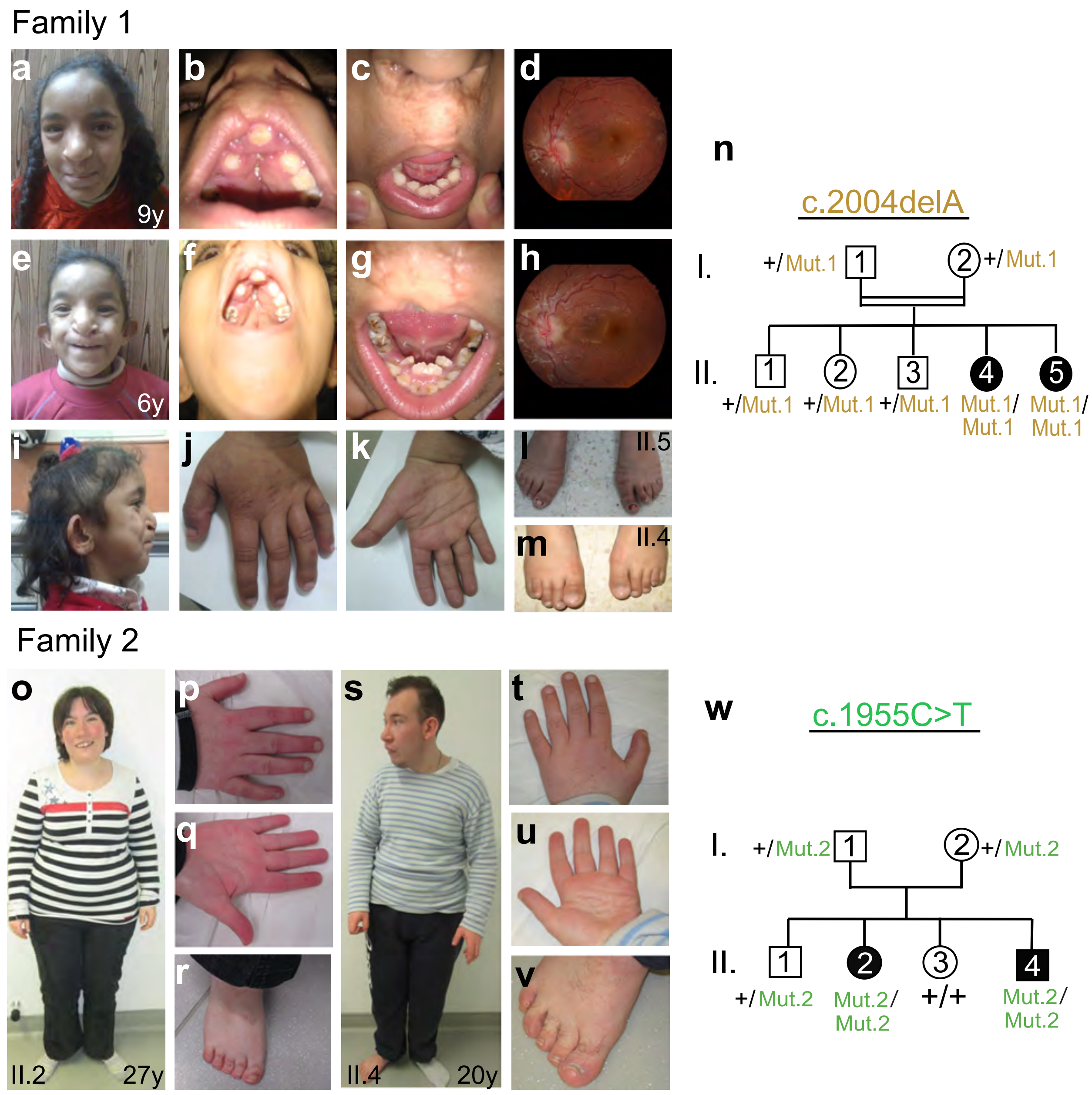

Supplementary Fig 1. Presentation of OFD2 Syndrome patients from Family 1 and Family 2. a-d, m Photographs of affected individual II.4 of Family 1 with common phenotype and orofacial dysmorphisms at different ages including: **a-c** Bilateral cleft lip and palate (corrected after multiple surgical operations), hypertelorism, broad nasal bridge, flat philtrum, dental abnormalities, low set ear and rough and sparse hair. **d** Crowded optic disc. m Clinodactyly of toes. **e-l** Photographs of affected individual II.5 of Family 1. **e-g** Bilateral cleft lip and palate, hypertelorism, broad nasal bridge, flat philtrum, dental abnormalities. **h** Crowded optic disc. **i** Low set ear and rough and sparse hair. **j, h** Broad and mild brachydactyly and single palmar creases. **l** Clinodactyly of toes. n Pedigree of Family I. The genotypes for the corresponding mutation are indicated below each individual. The c.2004delA mutation segregated with the disease in this family. (+/-) denotes the heterozygous alleles, and (-/-) denotes the homozygous mutant alleles. **o-v** Photographs of affected individuals II.2 and II.4 of Family 2 at 27 and 20 years old, respectively. **w** Pedigree of Family 2. The genotypes for the corresponding mutation are shown below each individual which confirm the segregation of the c.1955C>T mutation in this family. (+/+) denotes the homozygous wildtype alleles.

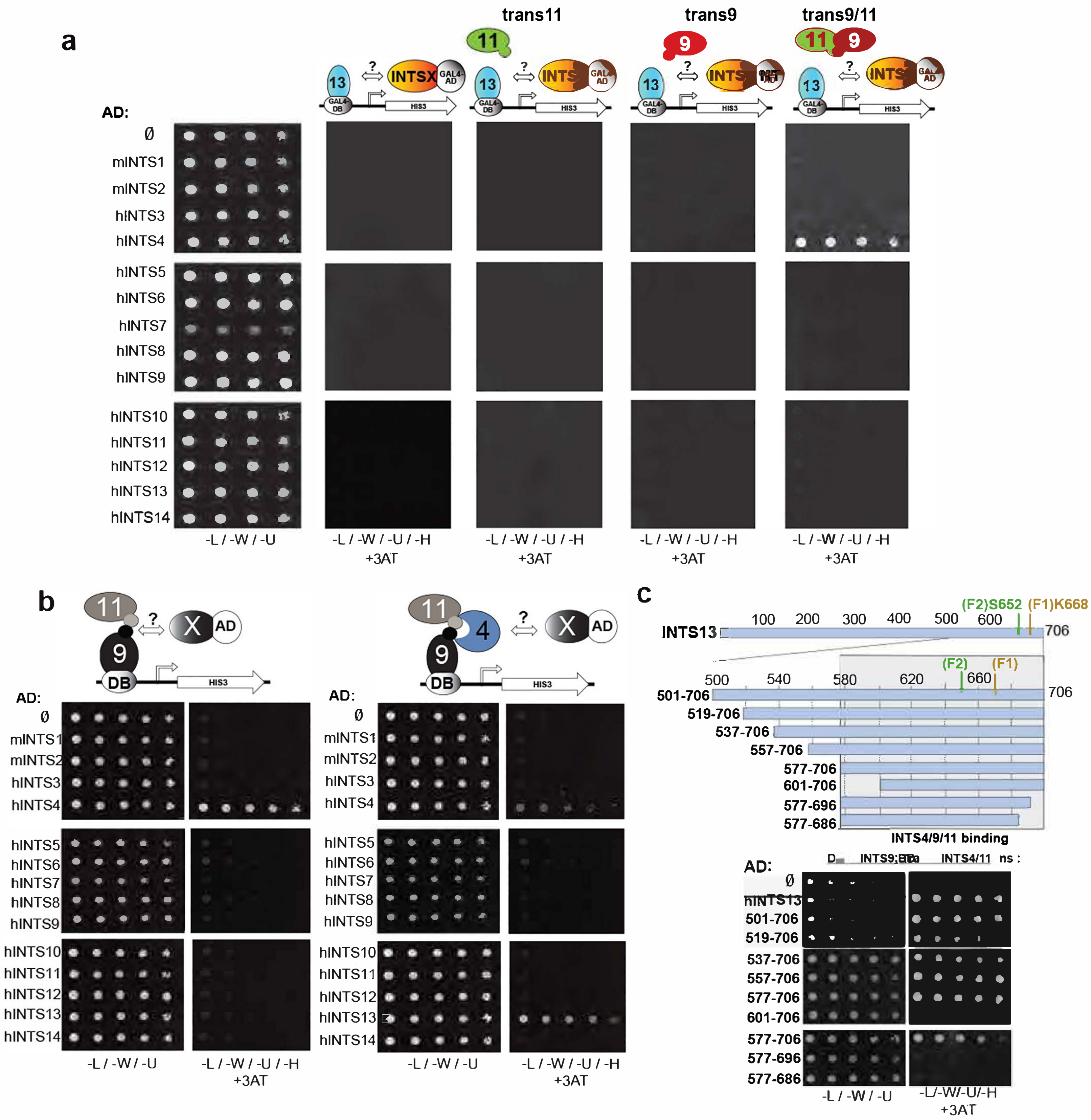

Supplementary Fig 2. The C-terminus of INTS13 interacts with the Integrator Cleavage Module. **a** Expanded modified yeast two hybrid assay from Fig 2a. Included here are tests where INTS11 or INTS9 were expressed in trans along with INTS13-binding domain and crossed with each Integrator subunit. No positive interactions were seen. **b** Left: Modified yeast two hybrid with INTS9 fused to the binding domain and INTS11 expressed in trans mated with yeast expressing each Integrator subunit fused to the activating domain to reconfirm that INTS4 interacts with the INTS9/11 heterodimer. Right: The same experiment with the addition of INTS4 expressed in trans to show that INTS13 interacts with the INTS4/9/11 heterotrimer. **c** Smaller portions (approximately 20 amino acids) of the INTS13 protein were removed from the necessary region for interaction shown in Fig 2d to determine that residues 577-706 were sufficient to interact with INTS4/9/11.

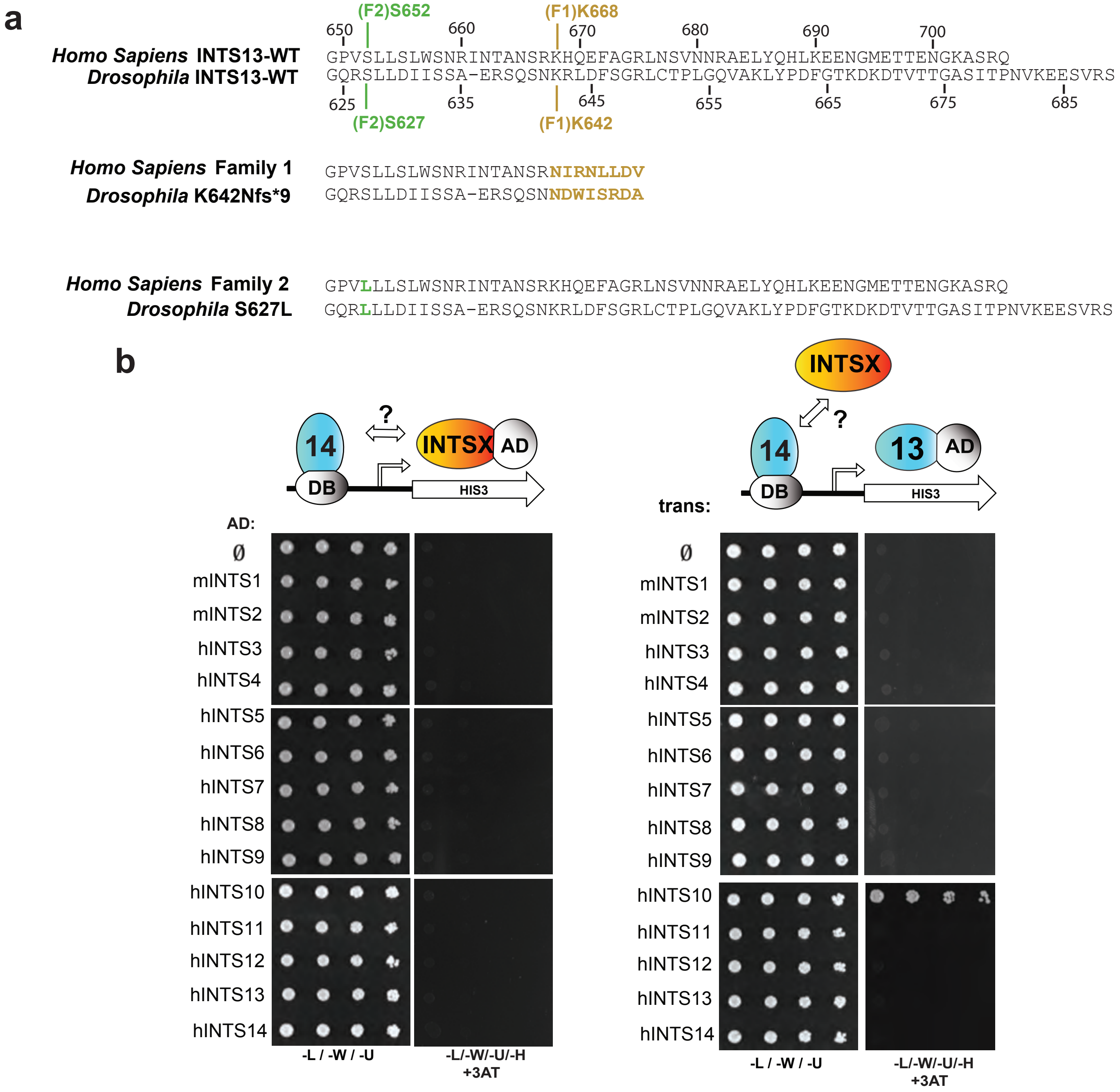

Supplementary Fig 3. INTS13 interacts with INTS10/14. **a** Partial protein alignment of the C-terminus of human and Drosophila INTS13 to show the orthologous mutations made to recapitulate the patient mutations in Drosophila cells. The location and altered residues are marked in brown for Family 1 and in green for Family 2. **b** Modified yeast two hybrid testing the interaction between INTS10/13/14. Left: INTS14 fused to the binding domain was crossed with each Integrator subunit fused to the activating domain, and no positive interaction was seen on selective media. Right: INTS13 fused to the activat-ing domain was crossed with yeast strains expressing INTS14-binding domain with each Integrator subunit in trans. A positive interaction was seen when INTS10 was expressed in trans.

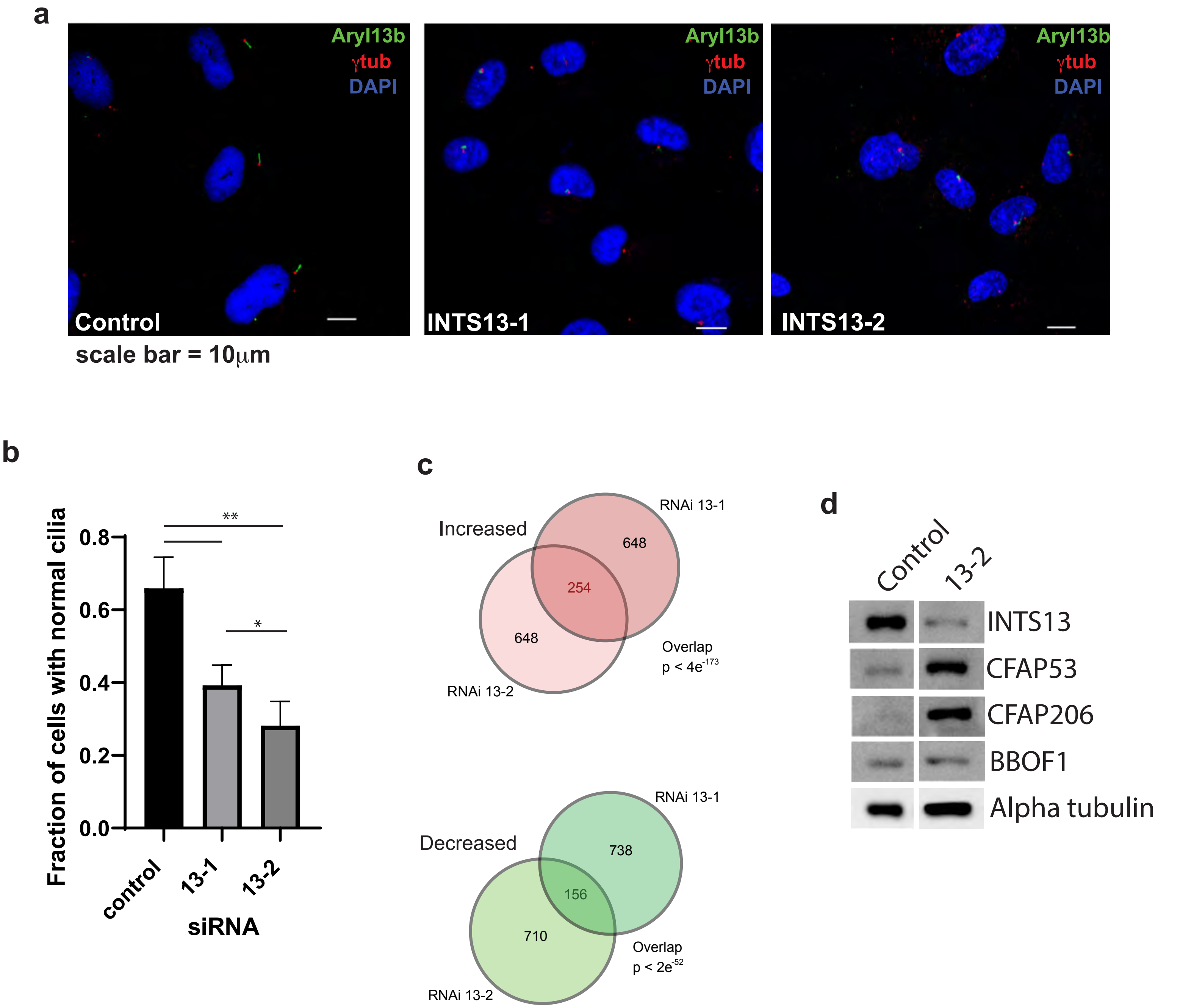

Supplementary Fig 4. RPE cells have disrupted cilia and ciliary gene expression upon INTS13 depletion. a Panned out images of RPE cells treated with either control siRNA, INTS13-1 siRNA, or 13-2 siRNA. Merged images are from nuclear staining with DAPI, Aryl13b, or γ-tubulin. Images are representative from dozens that were acquired and the RNAi-depletion was conducted in triplicate. **b**.Fraction of RPE cells with normal cilia as assessed by microscopy. Results shown are from triplicate samples where in each case, hundreds of cells were analyzed. Error bars represent SD from the mean. p value<0.0001 (**), p value<0.05 (*) using Tukey's multiple comparisons test **c**.Venn diagrams show the number of significantly upregulated or **b** downregulated genes for siRNA 13-1 and 13-2, and the genes in common between the two siRNAs as determined by RNA-seq. **d.** Western Blot analysis of lysates from RPE cells treated with either control siRNA or the more potent INTS13-2 siRNA; alpha-tubulin in used as a loading control. Note that the lanes were taken from the same blot at the same exposure but with the intervening lane removed.

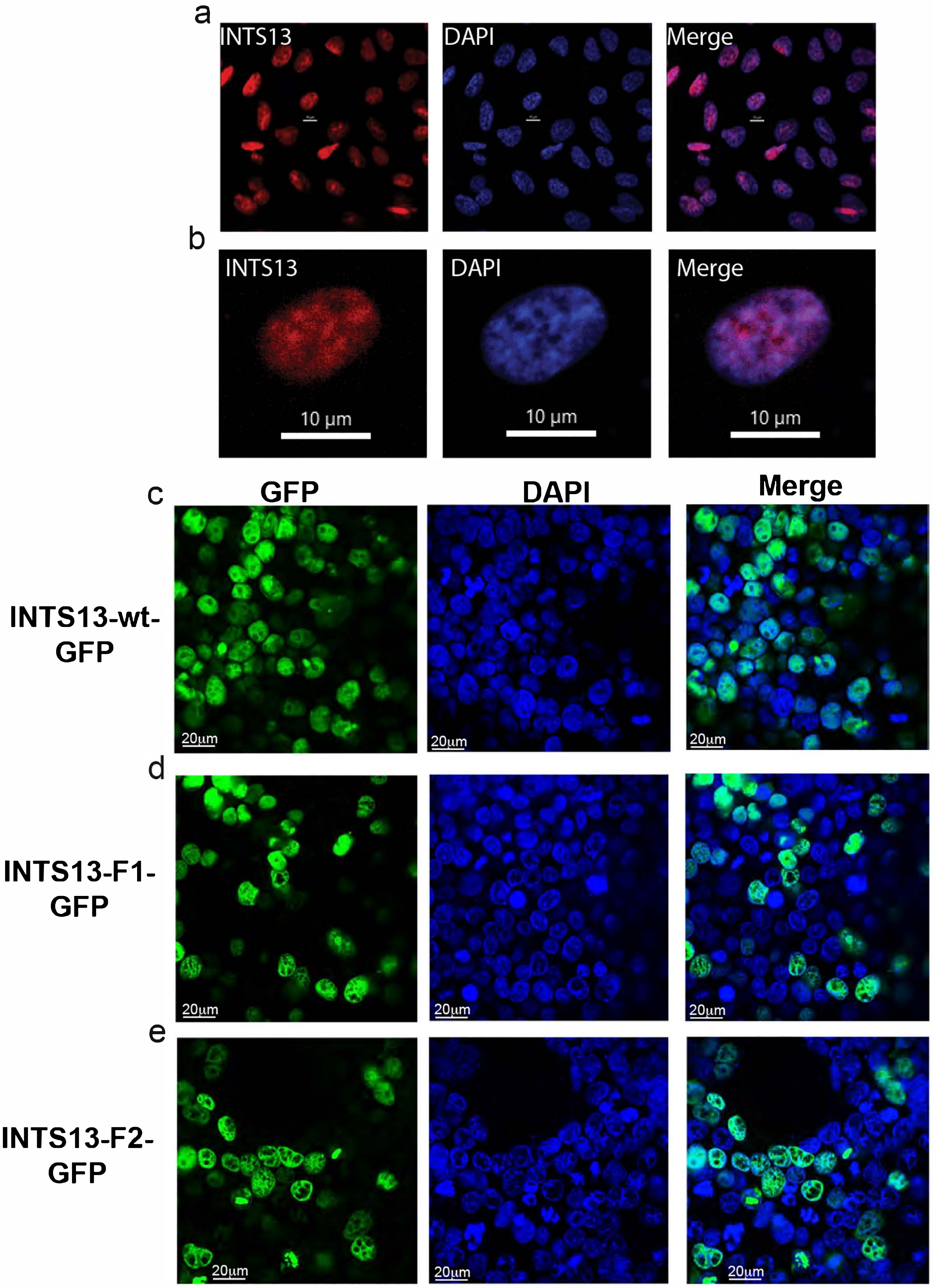

Supplementary Figure 5. **Integrator 13 localization in RPE and 293T cells is nuclear a** Localization of endogenous INTS13 in RPE cells as assessed by immunofluorescence. Scale bar is 10µm. Images are representative of multiple stainings of INTS13 (n>3) **b**. Selected image from panel A that is zoomed-in further. Scale bar is 10µm. **c.** GFP fluorescence in 293T cells transfected with INTS13-wt fused to GFP. The left panel is the image of cells viewed using a filter for GFP, the center panel depicts the nuclear as visualized using DAPI staining, and the right panel is the merger of the first two panels. **d.** same as in panel c except cells were transfected with IntS13-Family 1 mutant (F1) fused to GFP. **e.** Same as in panel c, except cells were transfected with INTS13-Family 2 mutant (F2) fused to GFP. Images are panned out to reflect multiple cells and are reflective of triplicate transfections done in parallel.

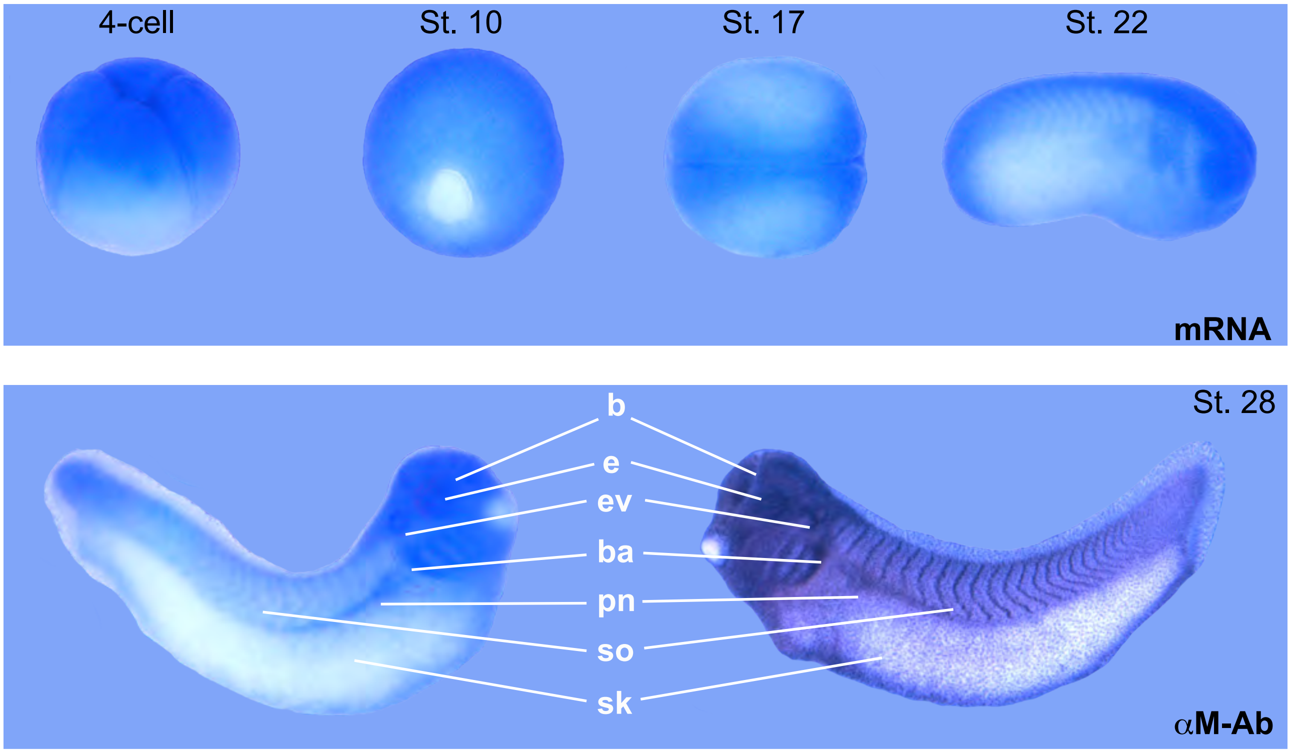

Supplementary Fig 6. Expression of *ints13* during Xenopus development. a Detailed expression pattern of *ints13* in Xenopus embryos analyzed by WISH and whole mount immunostaining. *ints13* has both maternal and zygotic contribution. At 4 cell stage, *ints13* transcripts are localized within the animal half. At stage 10, its expression covers the whole embryo except the blastopore. At stage 17, *ints13* is observed in neural tube and neural folds. b Lateral view, anterior is right stage 22 *ints13* starts to be enriched in brain, branchial arches (ba), pronephros (pn), eye vesicles (ev), ear (e), skin cells (sk) and somites (so). Lateral view, anterior is right of the embryo at stage 28, *ints13* expression is also detected in ear (e), skin cells (sk) and pronephros (pn). Correlation of transcripts localization of *ints13* protein at stage 28 by wholemount embryo antibody staining using a custom polyclonal INTS13 (α-M) antibody, Lateral view, anterior is left.
