# Supplementary material for: INTS13 variants causing a recessive developmental ciliopathy disrupt assembly of the Integrator complex
Source: Nat Commun. 2022 Oct 13;13:6054. doi: 10.1038/s41467-022-33547-8 (PMC9559116; doi:10.1038/s41467-022-33547-8)
Supplement: Supplementary file 2 — Description of Additional Supplementary Files [file 41467_2022_33547_MOESM2_ESM.pdf]

Description of Additional Supplementary Files

File Name: Supplementary Data 1
Description: **Clinical presentation of OFD type2-like syndrome patients.** All four affected family members from Family I or II were clinically analyzed. Descriptions of relevant clinical factors are provided. Hyperlinks to descriptors of all clinical terms are provided.

File Name: Supplementary Data 2
Description: **RNA-seq results from RPE-1 cells treated with either control siRNA or two distinct siRNA targeting INTS13.** In tab one, quantified gene expression changes for both INTS13 siRNA are provided in Tab 1. Fold change and p values of significance are provided and are the result of individual biological replicate knockdowns totaling triplicate samples (three control, three siRNA-1, and three siRNA-2). Normalized expression is also provided and this is the result of depth normalization. Tab 2 reflects a subset of the gene expression changes that are relevant to ciliogenesis through GO terms. This was a request of Reviewers during the evaluation process.

File Name: Supplementary Data 3
Description: **Mass Spectrometry of endogenous INTS13 from nuclear and cytoplasmic extracts.** All values represent normalized total spectra from either cell lysates (nuclear and cytoplasm mixed) or from nuclear extract alone that were either incubated with anti-INTS13 antibodies or control IgG antibodies. Highlighted rows represent Integrator subunits.

File Name: Supplementary Data 4
Description: **Sequences of all oligonucleotide primers used for cloning and morpholinos used for knockdowns.**

File Name: Supplementary Movie 1
Description: Recording of mucociliary epithelium of Xenopus uninjected tadpoles showing concerted beating of motile cilia.

File Name: Supplementary Movie 2
Description: Recording of mucociliary epithelium of Xenopus ints13 morphant tadpoles showing erratic or absent beating of motile cilia.
