# Supplementary material for: INTS13 variants causing a recessive developmental ciliopathy disrupt assembly of the Integrator complex
Source: Nat Commun. 2022 Oct 13;13:6054. doi: 10.1038/s41467-022-33547-8 (PMC9559116; doi:10.1038/s41467-022-33547-8)
Supplement: Supplementary file 9 — Reporting Summary [file 41467_2022_33547_MOESM9_ESM.pdf]

nature research

Corresponding author(s): Bruno Reversade and Eric J. Wagner

Last updated by author(s): 08 / 04 / 2022

Reporting Summary

Nature Research wishes to improve the reproducibility of the work that we publish. This form provides structure for consistency and transparency in reporting. For further information on Nature Research policies, see our Editorial Policies and the Editorial Policy Checklist.

Statistics

For all statistical analyses, confirm that the following items are present in the figure legend, table legend, main text, or Methods section.

n/a	Confirmed	
☐	☒	The exact sample size (*n*) for each experimental group/condition, given as a discrete number and unit of measurement
☐	☒	A statement on whether measurements were taken from distinct samples or whether the same sample was measured repeatedly
☐	☒	The statistical test(s) used AND whether they are one- or two-sided<br>*Only common tests should be described solely by name; describe more complex techniques in the Methods section.*
☒	☐	A description of all covariates tested
☐	☒	A description of any assumptions or corrections, such as tests of normality and adjustment for multiple comparisons
☐	☒	A full description of the statistical parameters including central tendency (e.g. means) or other basic estimates (e.g. regression coefficient) AND variation (e.g. standard deviation) or associated estimates of uncertainty (e.g. confidence intervals)
☐	☒	For null hypothesis testing, the test statistic (e.g. *F*, *t*, *r*) with confidence intervals, effect sizes, degrees of freedom and *P* value noted<br>*Give P values as exact values whenever suitable.*
☒	☐	For Bayesian analysis, information on the choice of priors and Markov chain Monte Carlo settings
☒	☐	For hierarchical and complex designs, identification of the appropriate level for tests and full reporting of outcomes
☒	☐	Estimates of effect sizes (e.g. Cohen's *d*, Pearson's *r*), indicating how they were calculated

Our web collection on statistics for biologists contains articles on many of the points above.

Software and code

Policy information about availability of computer code

Data collection	RNA-Seq data acquisition was via the Illumina NextSeq 500 platform using Software Suite v4.0.1. Data for qRT-PCR was acquired using Biorad CFX Manager (ver 3.1)
Data analysis	Exome sequencing and filtering was performed with publicly available as desrcibed in Materials and methods section.<br>RNA-Seq Data analysis was performed using open source software fastp (ver 0.14.1), Hisat2 (ver 2.1.0), DESeq2 (ver 1.23.10), and Rsubread (ver 1.30.9) as described in the Materials and methods section. Statistical analysis of qRT-PCR data was performed using the commercially available Graphpad Prism (ver 8.4.3) . LC-MS / MS analyzed by XCalbiber (ver 2.10) Images processed using Imaris Pro 9.3.0.

For manuscripts utilizing custom algorithms or software that are central to the research but not yet described in published literature, software must be made available to editors and reviewers. We strongly encourage code deposition in a community repository (e.g. GitHub). See the Nature Research guidelines for submitting code & software for further information.

Data

Policy information about availability of data

All manuscripts must include a data availability statement. This statement should provide the following information, where applicable:

- Accession codes, unique identifiers, or web links for publicly available datasets
- A list of figures that have associated raw data
- A description of any restrictions on data availability

-There is an RNA-seq dataset that supports Figure 5. We have submitted this dataset to GEO under: GSE154197, with the Reviewer token efitgsaazvuxfyp
-There are two sets of proteomics data in Figure 4. We have submitted these data to the ProteomeXchange Consortium via the PRIDE under the accession: PXD020593; Username: reviewer29920@ebi.ac.uk; password: C4KuWmEo

Field-specific reporting

Please select the one below that is the best fit for your research. If you are not sure, read the appropriate sections before making your selection.

☒ Life sciences ☐ Behavioural & social sciences ☐ Ecological, evolutionary & environmental sciences

For a reference copy of the document with all sections, see nature.com/documents/nr-reporting-summary-flat.pdf

Life sciences study design

All studies must disclose on these points even when the disclosure is negative.

Sample size

Cell culture experiments with results measured by qRT-PCR (mRNA expression levels in patient cells, cyclohexamide treatment, RNA-seq validation) were done in triplicate. Three biological replicates is an accepted sample size for qPCR statistics.
Immunofluorescence experiment quantification was done by counting the total cells in 6 separate cover slip samples for each condition. There was no sample size calculation performed but hundreds of cells were counted in each case. RNA sequencing was also done on biological triplicate samples for each condition with total depth of ~50M reads per replicate, which is an accepted sample size for this method.

Data exclusions

no data were excluded

Replication

Cell culture-based experiments were all reproduced. When quantifications are provided, then experiments were done with replicates. In the case of yeast 2H data are shown where positions of the Integrator subunits are switched to show reproducibility. For IPs, experiments were done using both WB and separately for MS to confirm findings. For KDs, two siRNA were used. For developmental phenotypes, two distinct model systems were used to demonstrate conserved outcomes.

Randomization

No experiments were done in this study that needed to be randomized.

Blinding

The IF experiments were done with blinding as the scientist who staged the cells (Mascibroda) was distinct from who quantified the primary cilium (SIngh). The samples were coded such that SIngh did not know the sample IDs.

Reporting for specific materials, systems and methods

We require information from authors about some types of materials, experimental systems and methods used in many studies. Here, indicate whether each material, system or method listed is relevant to your study. If you are not sure if a list item applies to your research, read the appropriate section before selecting a response.

Materials & experimental systems

n/a	Involved in the study
☐	☒ Antibodies
☐	☒ Eukaryotic cell lines
☒	☐ Palaeontology and archaeology
☐	☒ Animals and other organisms
☐	☒ Human research participants
X	Clinical data
☒	☐ Dual use research of concern

Methods

n/a	Involved in the study
☒	☐ ChIP-seq
☒	☐ Flow cytometry
☒	☐ MRI-based neuroimaging

Antibodies

Antibodies used

human INTS13/ASUN Rabbit polyclonal: Proteintech, #19892-1-AP
human ARL13B Mouse monoclonal: Proteintech, #66739-1-Ig
human gamma tubulin rabbit polyclonal: Proteintech, #15176-1-AP
human INTS1 rabbit: Bethyl, #A300-361A
human INTS3 rabbit polyclonal: Proteintech, #16620-1-AP
human INTS4 rabbit: Bethyl, #A301-296A
human INTS5 rabbit polyclonal: Proteintech, #14069-1-AP
human INT7 rabbit: Bethyl, #A300-271A
human INTS9 rabbit polyclonal: Atlas, #HPA066822
human INTS10 rabbit polyclonal: Proteintech, #15271-1-AP
human INTS11 rabbit polyclonal: Bethyl, #A301-274A
human INTS12 rabbit polyclonal: Proteintech, #16455-1-AP
human INTS14/C15orf44 rabbit: Bethyl, #A303-577A (discontinued: A303-576A is available)
human CFAP53 rabbit: RayBiotech, #144-64537-50
human C6orf165 (CFAP206) rabbit: Biorbyt, #orb156105
human CCDC176 (BBOF1) rabbit: Invitrogen, #PA5-70942
anti-rabbit donkey ECL Horseradish Peroxidase-linked secondary: GE Healthcare, Sigma Aldrich, #NA934V
Alexa Fluor 594 goat anti-rabbit secondary: Life Technologies, #A11037

	Alexa Fluor 488 goat anti-mouse secondary: Life Technologies, #A11029<br>anti-FLAG M2-Peroxidase (HRP) mouse monoclonal: Sigma, #A8592<br>antibodies to Drosophila proteins used in the Fig 3b western blot were made for the Wagner lab:<br>dIntS1 guinea pig (source was bacterially expressed & His-tagged protein, amino acids 1-150): Ezzedine et al, Mol. Cell. Biol., 2011<br>dIntS4 guinea pig (source was bacterially expressed & His-tagged protein, amino acids 1-150): Huang et al, 2020<br>dIntS8 guinea pig (source was bacterially expressed & His-tagged protein, amino acids 1-150): Huang et al, 2020<br>dIntS9 guinea pig (source was bacterially expressed & His-tagged protein, last 100 amino acids): Ezzedine et al, Mol. Cell. Biol., 2011<br>dIntS11 guinea pig(source was bacterially expressed & His-tagged protein, last 100 amino acids): Ezzedine et al, Mol. Cell. Biol., 2011<br>dIntS12 guinea pig (source was bacterially expressed & His-tagged protein, amino acids 1-150): Chen et al, J Biol. Chem., 2013
Validation	INTS13 Western blot from human cells: "HeLa cells were subjected to SDS PAGE followed by western blot with 19892-1-AP (ASUN antibody) at dilution of 1:4000 incubated at room temperature for 1.5 hours"<br>ARL13B immunofluorescence of human cells: "Immunofluorescent analysis of (4% PFA) fixed MDCK cells using 66739-1-Ig (ARL13B antibody) at dilution of 1:100 and Alexa Fluor 488-Conjugated AffiniPure Goat Anti-Mouse IgG (H+L)"<br>Gamma tubulin immunofluorescence of human cells: "IF results of gamma tubulin antibody (15176-1-AP, 1:100) with hTERT-RPE cells by Moshe Kim (laboratory of Dr. William S Trimble, University of Toronto). Cells were fixed in ice-cold methanol.<br>INTS1 Western blot from human cells: "Detection of human and mouse INT1 by western blot. Whole cells lysate (50 ug) from HeLa and HEK293T cells prepared using NETN lysis buffer. Affinity purified rabbit anti-INT1 antibody A300-361A (lot A300-361A-2) used for WB at 0.4 ug/ml. Chemiluminescence with an exposure time of 30 seconds."<br>INTS3 WB from human cells: "HEK-293 cells were subjected to SDS PAGE followed by western blot with 16620-1-aP (INTS3 antibody) at dilution of 1:500 incubated at room temperature for 1.5 hours"<br>INTS4 WB from human cells: "Detection of human and mouse INT4 by western blot an immunoprecipitation. Whole cell lysate from HEK293T cells (50 ug). Affinity purified rabbit anti-INT4 antibody A301-296A used fro WB at 0.4 ug/ml. Chemiluminescence with exposure time of 30 seconds."<br>INTS5 WB from human cells: "HeLa cells were subjected to SDS PAGE followed by western blot with 14069-1-AP (INTS5 antibody) at dilution of 1:500 incubated at room temperature for 1.5 hours."<br>INTS7 WB from human cells: "Detection of human INT7 by western blot. Nuclear extract from HeLa cells. Rabbit anti-INT7 antibody (Cat. No. 300-271A) used at 0.1 ug/ml. Chemiluminescence with an exposure time of 15 minutes."<br>INTS9 WB from human cells: "Western blot analysis in human cell line RT-4 and human cell line U-251 MG."<br>INTS10 WB from human cells: "Various lysates (MCF-7 and mouse kidney) were subjected to SDS PAGE followed by western blot with 15271-1-AP (INTS10 antibody) at dilution of 1:3000 incubated at room temperature for 1.5 hours."<br>INTS11 WB from human cells: "Detection of human and mouse INT11 by western blot. Whole cell lysate (50 ug) from HeLa cells prepared using NETN lysis buffer. Affinity purified rabbit anti-INT11 antibody A301-274A (lot A301-274A-4) used for WB at 0.1 ug/ml. Chemiluminescence with an exposure time of 3 minutes."<br>INTS12 WB from human cells: "HepG2 cells were subjected to SDS PAGE followed by western blot with 16455-1-AP (INTS12 anitbody) at a dilution of 1:1000 incubated at room temperature for 1.5 hours"<br>INTS14 WB from human cells: "Detection of human C15orf44 by western blot of immunoprecipitates. Whole cell lysate (1 mg for IP, 20% of IP loaded) from HeLa cells. Affinity purified rabbit anti-C15orf44 antibody A303-576A used fro IP at 6 ug/mg lysate. For blotting immunoprecipitated C15orf44, antibody BL12732 was used at 0.4 ug/ml. Chemiluminescence with an exposure time of 10 seconds."<br>CFAP53: product website shows a WB of the correct protein from both mouse and rat testis.<br>CCDC176 WB from human cells: "Western blot analysis of CCDC176 on human PANC1 cells. The sample was probed with a CCDC176 polyclonal antibody (Product # PA5-70942) using a primary antibody dilution of 1.0 µg/mL."<br>Drosophila antibodies (dIntS1, dIntS4, dIntS8, dIntS9, dIntS11, dIntS12) were validated using control and RNAi lysates

Eukaryotic cell lines

Policy information about cell lines

Cell line source(s)	ARPE-19 cells from ATCC, CRL-2302, lot #70004873 , High Five Cells from ThermoFisher, B85502<br>HEK293T cells from ATCC, CRL-3216<br>S2-DGRC clone 6 from DGRC (Drosophila Genomics Resource Center)
Authentication	Cell lines were not authenticated
Mycoplasma contamination	Potential Mycoplasma contamination was mitigated with Plasmocin
Commonly misidentified lines<br>(See ICLAC register)	none used

Animals and other organisms

Policy information about studies involving animals; ARRIVE guidelines recommended for reporting animal research

Laboratory animals	Xenopus laevis (both sexes, age is early in development as noted) and Drosophila melanogaster (males, under 10 days)
Wild animals	None
Field-collected samples	No field collected samples were used in this study
Ethics oversight	A*STAR-approved IACUC #201514 and #201555

Note that full information on the approval of the study protocol must also be provided in the manuscript.

Human research participants

Policy information about studies involving human research participants

Population characteristics	Four cases (1 male and 3 females) from 2 independent families In Family 1: two affected sisters born to a consanguineous Jordanian couple presented with a congenital disorder consistent with OFD2 (MIM252100. A homozygous frameshift (c.2004delA) mutation in INTS13 (MIM615079) was identified in the two affected sisters.<br>Family II: two affected siblings born to French parents. The 27-year-old affected girl and 20-year-old boy, presented with some overlapping phenotypes but also distinct features from those reported in the Jordanian patients. A homozygous missense mutation in INTS13 (NM_018164.2: c.1955C>T) was identified in the two affected individuals.
Recruitment	Family I: Patients were referred to the genetic clinic at the national center for diabetes endocrinology and genetics (NCDEG) for diagnosis and management (Amman, JORDAN)<br>Family II: Patients were referred to Imagine Institure (Paris, FRANCE).<br>Parental informed consent was obtained.
Ethics oversight	Ethical approval was obtained for both families with respective IRBs in France, Jordan and Singapore.<br>A*STAR IRB protocol #2019-087

Note that full information on the approval of the study protocol must also be provided in the manuscript.

Clinical data

Policy information about clinical studies

All manuscripts should comply with the ICMJE guidelines for publication of clinical research and a completed CONSORT checklist must be included with all submissions.

Clinical trial registration	not applicable
Study protocol	not applicable
Data collection	not applicable
Outcomes	not applicable
